# Lipophilic Prodrug of Methotrexate in the Membrane of Liposomes Promotes Their Uptake by Human Blood Phagocytes

**DOI:** 10.32607/actanaturae.10946

**Published:** 2020

**Authors:** D. S. Tretiakova, S. V. Khaidukov, A. A. Babayants, I. S. Frolova, O. N. Shcheglovitova, N. R. Onishchenko, E. L. Vodovozova

**Affiliations:** Shemyakin–Ovchinnikov Institute of Bioorganic Chemistry, Russian Academy of Sciences, Moscow, 117997 Russia; Gamaleya National Research Center for Epidemiology and Microbiology, Ministry of Healthcare of the Russian Federation, Moscow, 123098 Russia

**Keywords:** methotrexate, lipophilic prodrug, liposomes, leukocytes, phagocytosis, flow cytometry

## Abstract

Previously, we showed that incorporation of methotrexate (MTX) in the form of a lipophilic prodrug (MTXDG) in 100-nm lipid bilayer liposomes of egg phosphatidylcholine can allow one to reduce toxicity and improve the antitumor efficiency of MTX in a mouse model of T-cell leukemic lymphoma. However, in our hemocompatibility tests *in vitro*, MTX liposomes caused complement (C) activation, obviously due to binding on the liposome surface and fragmentation of the C3 complement factor. In this work, we studied the interactions of MTX liposomes carrying stabilizing molecules phosphatidylinositol (PI), ganglioside GM1, or a lipid conjugate of* N*-carboxymethylated oligoglycine (CMG) in the bilayer with subpopulations of human blood leukocytes. Liposomes labeled with BODIPY-phosphatidylcholine were incubated with whole blood (30 min and 1 h, 37°C), blood cells were lysed with a hypotonic buffer, and the fluorescence of the liposomes bound but not internalized by the leukocytes was quenched by crystal violet. Cell suspensions were analyzed by flow cytometry. Incorporation of MTXDG dramatically enhanced the phagocytosis of liposomes of any composition by monocytes. Neutrophils consumed much less of the liposomes. Lymphocytes did not accumulate liposomes. The introduction of PI into MTX liposomes practically did not affect the specific consumption of liposomes by monocytes, while CMG was likely to increase the consumption rate regardless of the presence of MTXDG. The GM1 ganglioside presumably shielded MTX liposomes from phagocytosis by one of the monocyte populations and increased the efficiency of monocyte uptake by another population, probably one expressing C3b-binding receptors (C3b was detected on liposomes after incubation with blood plasma). MTX liposomes were shown to have different effects on TNF-α production by activated leukocytes, depending on the structure of the stabilizing molecule.

## INTRODUCTION

The cytostatic agent methotrexate (MTX) is registered by the World Health Organization on the List of Essential Medicines [1]. MTX is a folic acid antimetabolite; it is widely used in the treatment of solid tumors, hematological malignancies, and autoimmune pathologies, such as rheumatoid arthritis, where it remains the drug of choice [2, 3]. However, the use of MTX is limited by both its high general toxicity and the development of cellular resistance, which is mainly associated with impaired transport of MTX into the cells that has to do with mutations and decreased activity of the transporter protein of reduced folate and antifolate analogues (reduced-folate carrier, RFC) [3, 4]. Passive transmembrane transfer of the MTX polar molecule is difficult. It is possible to overcome this barrier and improve the pharmacological properties of MTX by encapsulating it in a nanosized carrier that would protect the drug from premature interaction with biomolecules in the bloodstream and deliver it to the cell via pinocytosis. In the last decade, nanoscale MTX delivery systems, including polyamide dendrimers, polymer nanogels, nanocapsules of triglycerides and surfactants, etc., have been intensively studied [5, 6, 7, 8]. The MTX conjugates with serum albumin and liposomes are recognized as the most promising ones for systemic administration into the body [5]. Thus, intravenous injections of PEGylated (i.e., coated with polyethylene glycol, PEG) MTX-encapsulating liposomes bearing a targeting peptide significantly improved the state of mice with experimental encephalomyelitis in [8]. However, the drug load in most of the presented nanoscale systems is very low. At the same time, for oncological diseases, doses of MTX that are several times higher (even in the low-dose therapy regimen) than in the case of anti-inflammatory therapy are required.

**Fig. 1:**
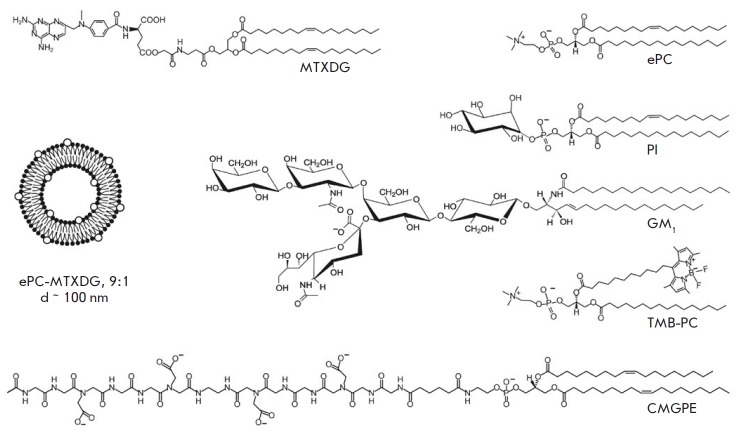
Schematic representation of a liposome loaded with a lipophilic prodrug of methotrexate (MTXDG) and the chemical structures of liposome components: CMGPE, peptide–lipid conjugate; TMB-PC, BODIPY-labeled phosphatidylcholine. Representative structures of egg phosphatidylcholine (ePC), soybean phosphatidylinositol (PI), and ganglioside GM1 from a bovine brain are also presented

Methotrexate cannot be encapsulated in nanosized liposomes using the remote loading technique, an effective method used for weak amphipathic acids or bases, for example anthracycline antibiotics, such as doxorubicin [9]. In the case of passive encapsulation, loading of 100-nm liposomes with a water-soluble drug does not exceed 2–3 mol. % to total lipids. We have developed liposomes carrying 10 mol. % MTX in the form of a lipophilic prodrug, a dioleoyl glyceride ester conjugate at the α-COOH glutamate residue (MTXDG, *Fig. 1*), in a lipid bilayer made of natural phospholipids [10]. In a culture of RFC-deficient T-lymphoblastoid cells, such MTX liposomes overcome resistance to MTX [11]. In a mouse model of acute T-cell leukemic lymphoma, MTX liposomes inhibited tumor growth more efficiently than intact MTX and were less toxic [12].

Thanks to their exceptional bio- and hemocompatibility, liposomes became the first drug delivery systems used in clinical practice [13-15]. However, the liposomes introduced into the bloodstream, like other particles similar in size to viruses, primarily encounter leukocytes and undergo phagocytosis [16, 17]. Premature elimination of drugs from the bloodstream by myeloid cells is the main barrier to their delivery by nanocarriers to target organs and tissues. To stabilize nanosized liposomes in the bloodstream, screening by highly hydrophilic PEG chains has been developed [18]. However, PEGylation, as well as coating with other polymers, turned out not to interfere with non-specific protein binding; this can cause infusion reactions of varying severity, up to anaphylactic shock, associated with the activation of the complement (C) [19-22]. Protein corona – a complex layer of proteins and lipoproteins – is formed within a few seconds upon contact of nanoparticles, including liposomes, with blood plasma [23, 24]. Opsonization of a nanocarrier by C proteins promotes its recognition by the receptors of immunocompetent cells.

In functional hemocompatibility tests, liposomes loaded with MTXDG did not affect the main human blood cells, i.e. red blood cells and platelets, but they caused moderate impairment of the blood coagulation system and activated C [25]. Indeed, after incubation of MTX liposomes in plasma, fragmentation of the central C component, the C3 protein, was observed, an indication of activation of the C cascade [26, 27]. Liposomes of similar phospholipid composition carrying the diglyceride conjugate of another cytotoxic agent, melphalan, did not cause such effects [25, 26, 27]. That is, the surface properties of liposomes affect the composition (and quantity) of the plasma proteins bound, which determines the inertness of liposomes in the bloodstream or their potential to cause infusion reactions. In this work, using flow cytometry, we investigated the effect of amphiphilic screening molecules (*Fig. 1*), other than PEG–lipid conjugates, in the membrane of MTX liposomes on the interactions with subpopulations of human leukocytes in whole blood. Since these interactions are primarily mediated by C components and immunoglobulins G, we compared the levels of binding of the C3 protein and IgG to liposomes of various compositions. In addition, the effect of the composition of liposomes on the manifestations of innate immunity was studied by the example of the pro-inflammatory cytokine TNF-α production.

## EXPERIMENTAL

**Materials**

Diglyceride conjugate of methotrexate MTXDG was synthesized as described in [28]. Egg yolk phosphatidylcholine (ePC) from Lipoid GmbH (Heidelberg, Germany) was used; raw soybean phosphatidylinositol (PI) provided by Lipoid was purified by column chromatography. 1,3,5,7-Tetramethyl-BODIPY-labeled phosphatidylcholine (TMB-PC) was synthesized [29] and kindly provided by Dr. I.A. Boldyrev (IBCh RAS). Phospholipid conjugate of carboxymethylated oligoglycine (CMGPE) was synthesized and kindly provided by Dr. A.B. Tuzikov (IBCh RAS). GM1 ganglioside was isolated from the brain of cattle and was kindly provided by Dr. I.I. Mikhalev (IBCh RAS). Sepharose CL-4B was from Pharmacia (USA), ethylenediaminetetraacetic acid (EDTA) and the rest of the reagents were from Sigma and Flow Laboratories (USA). The solvents were purified by standard methods; evaporation was carried out under vacuum at temperatures below 40°C. Buffers were prepared in bidistilled water (H_2_O_dd_): PBS, phosphate-buffered saline (KH_2_PO_4_, 0.2 g/L; NaH_2_PO_4_ × 2H_2_O, 0.15 g/L; Na_2_HPO_4_, 1.0 g/L; KCl, 0.2 g/L; NaCl, 8.0 g/L) with 1 mM EDTA, pH 7.4; lysis buffer (NH_4_Cl, 155 mM; NaHCO_3_, 12 mM; EDTA, 0.1 mM).

Blood samples of healthy volunteer donors were collected in test tubes over lithium heparin as an anticoagulant (Vacuette, Greiner Bio-One, Germany) and stored in the dark at a temperature of 20–22°C for no more than 8 h.

**Liposome preparation**

Liposomes (monolamellar vesicles) were obtained by extrusion as described previously [11, 12, 26, 27]. Briefly, mixtures of phospholipids, MTXDG, and other membrane components at the required ratios were evaporated in round-bottom tubes from solution in chloroform–methanol (2 : 1) on a rotary evaporator and kept for 40 min at 7 Pa (INEY-4 freeze dryer; Institute of Biological Instrumentation, RAS, Russia). The compositions of lipid films (mol/mol) were as follows: ePC–MTXDG, 9 : 1; ePC–MTX-DG–PI, 8 : 1: 1; ePC–MTXDG_–_GM1, 8 : 1: 1, ePC–MTXDG–CMGPE, 8 : 1: 1, as well as the empty (prodrug-free) samples ePC, ePC_–_PI, 9 : 1; ePC_–_GM1, 9 : 1, ePC–CMGPE, 9 : 1. All compositions also contained 1 mol. % TMB-PC. The lipid films were hydrated for 2 h at room temperature in 0.3 ml of PBS, shaken to obtain a suspension, then subjected to 10 cycles of freezing/thawing (liquid nitrogen/+ 40°C) and extruded 10 times through polycarbonate membrane filters (Nucleopore, USA) with a pore size of 100 nm using the Mini-extruder (Avanti Polar Lipids, USA). Concentration of MTXDG in liposome dispersions was determined by spectrophotometry after liposome disruption by 20-fold dilution in ethanol (λ_max_ 302 nm, ε ~ 25000 M^–1^ cm^–1^). Liposome size was monitored using the90+Particle Analyzer (Brookhaven Instruments Corp., USA; helium-neon laser, λ 633 nm, 90°); the MTX liposome diameters ranged from 100 to 110 nm.

**Incubation of liposomes with whole blood, preparation of samples for cytometry**

An aliquot of 5 μl of a 10-mM liposome sample was added to 100 μl of whole blood, mixed, and incubated at 37°C for 30 or 60 min. As a control, 5 μl of PBS was added to 100 μl of whole blood. After incubation, the samples were diluted with 3 ml of cold PBS (+4°C) to stop phagocytosis, intensively stirred, and centrifuged for 10 min at 250 *g*. The supernatant was discarded, 1 ml of cold lysing buffer (+4°C) was added, and the mixture was stirred and left for 1 h in the dark at +4°C. Immediately before the measurements, to quench the fluorescence on the cell surface, an aliquot of an aqueous solution of crystal violet was added to a final concentration of 0.1 mg/ml and the sample was actively stirred. Samples were prepared in duplicates.

**Cytometry**

Stained cell measurements were performed on a Cytomics FC500 flow cytometer (Beckman Coulter, Florida, USA). Based on preliminary experiments, the research protocol, including the choice of the analysis zone, fluorescence sensitivity (photomultiplier voltage) and voltage across the light scattering channels, was standardized. This protocol was used later in all experiments conducted on the blood of various donors. Based on the control experiments (0 min incubation), the boundaries of positive and negative cells were defined on the fluorescence distribution histograms so that the main pool of negative cells remained in the first decade of the logarithmic scale. The target peripheral blood leukocyte populations (lymphocytes, monocytes, and granulocytes) were detected by introducing logical constraints into cell distribution histograms for small-angle (forward scatter) and lateral (side scatter) light scattering using standard FACS analysis criteria [30]. Each population was individually analyzed using fluorescence of at least 105 cells. The control experiment showed that identification of blood cell subpopulations by morphological parameters yields the same results as staining with the CD45 leukocyte marker. The collected data were processed using the CXP analysis software package (Beckman Coulter, USA).

**Liposome incubation in plasma and isolation of liposome–protein complexes**

Plasma was separated from whole blood by centrifugation for 10 min at 2000 *g *(Jouan BR4i, Thermo Fisher Scientific, USA). The supernatants were transferred to fresh tubes, and residual platelets and other cells were separated by centrifugation (30 min at 2000 rpm) at room temperature (Sorvall RT 7 Plus, Thermo Fisher Scientific, USA). The supernatants were combined, frozen in liquid nitrogen N2, and stored at –70°C. Plasma aliquots were thawed immediately prior to the experiments. Liposomes (90 μl) were incubated with 360 μl of plasma at 37°C with gentle stirring in Eppendorf tubes (Germany) (1.5 ml, 15 min), unless otherwise indicated. As a negative control, a plasma sample with PBS (4 : 1) was prepared. The mixture was applied to a Sepharose CL-4B column (~1.1 × 19 cm), PBS was eluted, and fractions of ~400 μl were collected. Aliquots of the fractions (80 μl) were mixed with 400 μl EtOH, centrifuged for 10 min at 9000 g (11000 rpm, Eppendorf centrifuge), and the content of MTX-DG in the supernatants was analyzed by spectrophotometry. In parallel, 100 μl of each fraction was taken to determine the amount of protein. Isolation of liposome–rotein complexes was carried out at least twice for each liposome sample.

Protein was determined using the modified Lowry method [31]. Reagent C was prepared immediately before use: reagent B (4% CuSO4 in H_2_Odd) was added to reagent A (2% Na_2_CO_3_ + 0.4% NaOH + 0.26% NaK tartrate + 1% SDS in H_2_Odd) 100 : 1 (v/v). To 100 μl of the analyzed solution, 300 μl of reagent C was added, stirred, and after 10 min, 30 μl of the Folin reagent diluted with H_2_Odd 1 : 1 was added. After 60 min, the absorbance was measured at 660 nm. The control sample contained 100 μl of PBS.

**Delipidization of pooled protein fractions and PAGE**

Delipidization was performed as described in [32]. To 100 μl of the combined fractions of liposome–rotein complexes, 400 μl of chilled MeOH was added, mixed, and centrifuged for 3 min at 9000 *g*. Then 200 μl of CHCl_3_ was added to the solution, shaken, and centrifuged for 3 min at 9000 *g*. After adding 300 μl of H_2_Odd to the mixture, shaking, and centrifuging (4 min at 9000 *g*), phase separation was observed and the protein concentrated at the interface. Approximately 700 μl of the upper phase was discarded, and 300 μl more MeOH was added to the residue; the mixture was stirred and centrifuged for 4 min at 9000 *g*. The supernatant was decanted, leaving ~30–50 μl, which were evaporated to dryness on a rotary evaporator. Then, 36 μl of the sample buffer (0.075 M Tris-HCl, pH 6.8, 10% glycerol, 2% SDS, 5% β-mercaptoethanol, 0.01% bromphenol blue) was added to the samples; the mixtures were stirred and kept in a water bath (90–95°C) 2 × 2 min, with active mixing. Lammley electrophoresis [33] was performed in a 6% concentration and 12% separating gel on a Helicon VE-2M device (Russia): pre-electrophoresis, 6 min, 10 mA; concentrating electrophoresis, 20 min, 18 mA; separating electrophoresis, 40 min, 28 mA. The proteins were visualized by silver staining [34]. Electrophoregrams were analyzed using the ImageJ software. To correlate the molecular weights of the protein bands, the Thermo Scientific Prestained Protein Molecular Weight Marker kit (Thermo Fisher Scientific, USA) was used.

## EXPERIMENTAL PROCEDURES

**Immunoblotting**

The proteins were transferred onto the Immobilon-P (Merck Millipore, Germany) polyvinylidene fluoride membrane using a semi-dry transfer device (Semidry; Helicon, Russia) for 30–40 min at a voltage of 35 V. After the transfer was completed, the membrane was rinsed with H_2_Odd, washed with TBS buffer (NaCl, 4.39 g; Tris, 3.03 g; H_2_Odd, 500 ml), pH 7.97, and incubated in a 5% low-fat dry milk dispersion in TBS supplemented with 0.1% Tween 20 (TBS/T) for 1 h at room temperature to prevent nonspecific adsorption. The membrane was then washed with TBS/T (3 × 5 min) and incubated overnight at 4°C with primary antibodies to the component C3 of human C (goat antibodies, ComplementTech, USA) or with antibodies to human immunoglobulin G conjugated to horseradish peroxidase (goat antibodies, Santa Cruz Biotechnology, USA) in a 0.5% solution of bovine serum albumin. The membrane was washed with TBS/T (15 min and 3 × 5 min). In the case of blotting with anti-C3 antibodies, the membrane was further incubated with secondary IgG conjugated to horseradish peroxidase (rabbit anti-goat IgG antibodies, Jackson ImmonoResearch, USA) then washed again with TBS/T (15 min, 2 × 5 min) and TBS (5 min). Immunodetection was performed using the Clarity ™ ECL Western Blotting Substrate reagent (Bio-Rad, USA) and the VersaDoc 4000 system (Bio- Rad).

**Testing the production of tumor necrosis factor alpha (TNF-α) by activated leukocytes**

Donor blood was diluted with the RPMI-1640 medium (Gibco) to a final leukocyte concentration of 1 × 106/ml and added into 24-well plates containing 0.9 ml of the suspension. Leukocytes were activated with phytohemagglutinin (PHA) (Sigma) by the addition of 100 μl of the PHA solution to the wells to a final concentration of 10 μg/ml. In the control wells with the cells, 100 μl of the RPMI-1640 medium was added. After 4 h of exposure in a cell incubator at 36.8°C and 5% CO_2_, 50 μl of methotrexate solutions were added to the wells to a final concentration of 50 μM, MTX liposomes to a final concentration of MTXDG of 50 μM, and MTXDG-free liposomes to a final lipid concentration similar to that in the wells with MTX liposomes, 500 μM. Then, 50 μl of PBS was added to the PHA control wells and to the control wells with the cells. After incubaiton in a CO_2_ incubator for 4 h, samples of the culture medium were taken, frozen, and stored at –30°C for subsequent testing. In the samples, TNF-α was determined by ELISA using the test system created by VectorBest (Russia) following the manufacturer’s instructions. The results of two independent experiments, each in duplicates, were obtained using the Anthos 2020 microplate photometer (Biochrom Ltd, Cambridge, UK) at a wavelength of 540 nm.

## RESULTS AND DISCUSSION

Phosphatidylinositol (PI), ganglioside GM1, or a phospholipid conjugate of carboxymethylated oligoglycine (CMGPE) were inserted into the MTX liposomes as components capable of shielding the membrane from opsonization (Fig. 1). According to [35], incorporation of PI into the bilayer reduces the liposome uptake by the cells of the reticuloendothelial system. This effect can be explained by the negative charge of the phospholipid and a relatively large head group, as well as the steric hindrances created by the highly hydrated myoinositol moieties on the surface of the liposomes [36]. The GM1 ganglioside in the liposomes increased their circulation lifetime even more than PI due to the voluminous and rigid, negatively charged pentasaccharide residue [37]. In our experiments, liposomes with a diglyceride conjugate of melphalan containing the indicated natural lipids or the new CMGPE compound in the egg phosphatidylcholine membrane turned out to be significantly more stable in blood plasma than similar liposomes with a PEG–lipid conjugate [38].

**Fig. 2:**
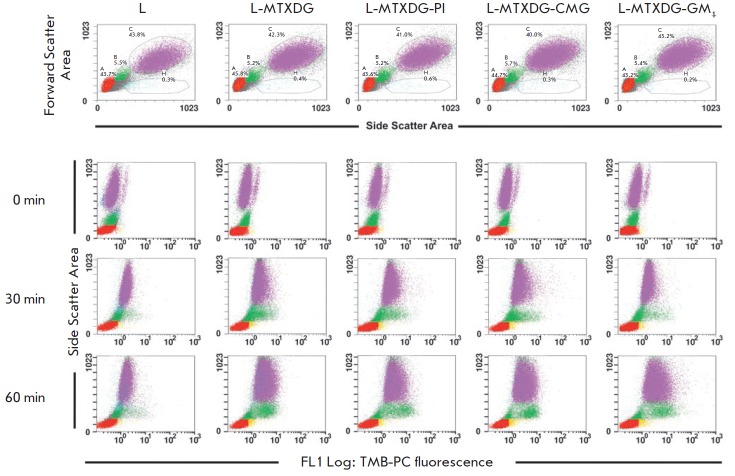
FACS histograms of blood samples after incubation with liposomes and erythrocyte lysis. Upper panel: distribution of peripheral blood cells in samples without liposomes; A, lymphocytes; B, monocytes; C, neutrophils; the area of dead cells is highlighted below. Panels 0, 30, 60 min: fluorescence of phagocytized liposomes “gated” by subpopulations of leukocytes after incubations for 0, 30, 60 min (a dye was added to quench the fluorescence of the liposomes adsorbed on the surface of cells; yellow zones are cells with incompletely quenched liposome fluorescence on the surface). TMB-PC liposomes of the following compositions were used: ePC (sample L); ePC–MTXDG, 9 : 1 (sample L-MTXDG); ePC–MTXDG–PI, 8 : 1 : 1 (sample L-MTXDG-PI ); ePC–MTXDG–CMGPE, 8 : 1 : 1 (sample L-MTXDG- CMG), ePC–MTXDG–GM1, 8 : 1 : 1 (sample L-MTXDG-GM1)

The use of cytometry to study the interactions between the liposomes and subpopulations of blood leukocytes is described only in a few publications [39, 40, 41, 42]; in [41] and [42], the liposomes were incubated with isolated neutrophils or mononuclear cells, respectively, and not with whole blood. In [39], blood was incubated with “solid-phase” PEGylated liposomes for 3 h; in [40], 5 h. Then red blood cells were lysed in a hypotonic buffer and FACS analysis was performed, detecting the total fluorescence of bound and consumed liposomes. In our case, the incubation protocol was changed. To exclude the liposomes adsorbed but not phagocytized by the cells from registration, a vital dye crystal violet was used as a fluorescence quencher [43]. The critical stages of the design of the experiment were the selection of the incubation time and conditions for the lysis of red blood cells while maintaining the integrity of the neutrophils. Taking into account the time required to prepare a sample for cytometry (after incubation of liposomes with blood) and a storage time of whole blood of no more than 8 h, no more than three liposome variants, each in duplicates, could be analyzed in one experiment. *Figure 2* shows representative FACS histograms of the uptake of TMB-PC-labeled liposomes of various compositions by blood leukocyte subpopulations. Samples of donated blood were obtained with an interval of one day; the number of cells in the subpopulations varies slightly.

**Fig. 3:**
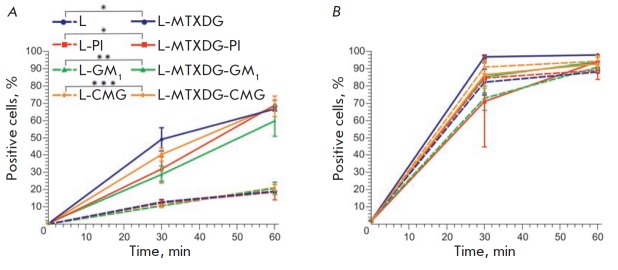
Kinetics of liposome uptake by monocytes (*A*) and neutrophils (*B*) of human whole blood *ex vivo*. Mean values ± SE of two independent experiments are presented, each in two repeats; the values of * *p* < 0.04, ** *p* < 0.02, and *** *p *= 0.002 are given for data obtained after 30 min of incubation

**Fig. 4:**
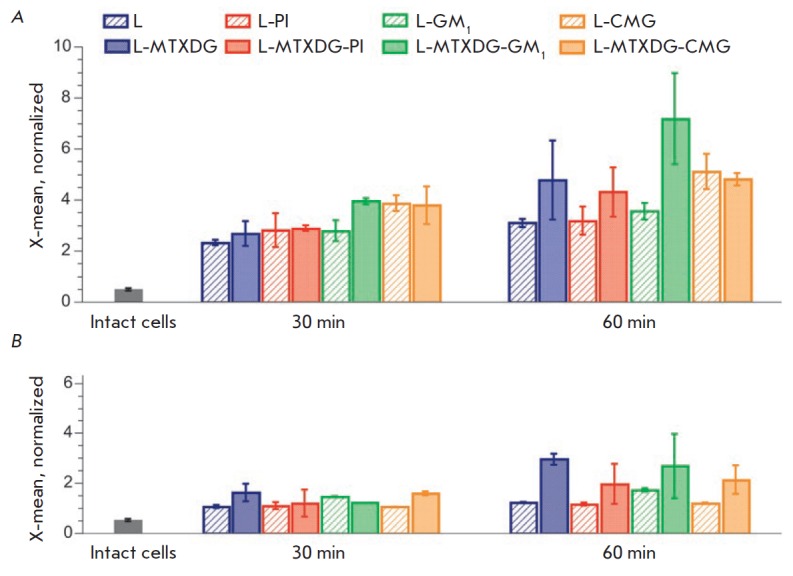
The average fluorescence intensity (X-mean) of the absorbed liposomes for positive populations of monocytes (*A*) and neutrophils (*B*). Mean values ± SE of two independent experiments are presented, each in two repeats

Obviously, lymphocytes do not accumulate liposomes, which agrees with the lack of the ability to phagocytize in these cells and is consistent with the modern concept that the majority of nanoparticles in the bloodstream are phagocytized by monocytes and neutrophils [17, 39, 40]. A small population of cells (0.6–1.3%) outlined in the neutrophil zone of the histogram (*Fig. 2*, 0 min panel) is represented by eosinophils, which are not professional phagocytes, yet are able to absorb small particles and cells (microphagocytosis), and, therefore, liposomes. However, the content of eosinophils in blood is so low that their contribution to the uptake of liposomes is negligible compared to the main populations of phagocytes. In *Fig. 3*, the results of the FACS analysis are presented as growth of the percentage of cells that accumulate liposomes during 1 h of incubation. Given the scatter of the data between the repeats (approximately 10%), it can be concluded that MTX liposomes accumulate faster in monocytes than liposomes without prodrugs, regardless of the presence of certain protective amphiphilic molecules; that is, MTXDG molecules in the membrane contribute to the acceleration of liposome uptake by a population of monocytes. When CMGPE, PI, or GM1 was introduced into the membrane, a tendency to slower phagocytosis of MTX liposomes by monocytes was observed (*Fig. 3*). But by the 60th minute, all differences between the samples of MTX liposomes were leveled: approximately 60–70% of monocytes and about 98% of neutrophils participated in their phagocytosis. At the same time, the intensity of liposome uptake by neutrophils (average fluorescence intensity, X-mean) was significantly lower than that of monocytes (*Fig. 4*): taking into account that the neutrophil population is approximately 7-fold larger than that of monocytes, each neutrophil internalized about 15 times less liposomes than a monocyte. The low level of liposome phagocytosis by neutrophils and the high rate at which it achieved a plateau can be attributed to the fact that the leading role of these cells is to protect against bacterial, and not viral, infections [44] (liposomes are comparable in size to viral particles).

Endocytosis of liposomes by blood phagocytes is mediated by the receptors of plasma proteins associated with liposomes. The main receptors for liposome uptake are considered to be receptors for the constant regions of IgG (FcγR I–III) and receptors for the complement factor fragments C3b and iC3b (CR1 and CR3, respectively) [24]. The C3b component has not been detected on the surface of liposomes without MTXDG by immunoblotting of liposome-associated proteins [26, 27]; i.e., its quantity was negligible, while in the case of MTX liposomes (L-MTXDG-PI), bands of its fragments could be observed. These data and the different levels of uptake of MTXDG-loaded and MTXDG-free liposomes by the cells (60 min incubation, *Fig. 4*, except for CMGliposomes) are consistent with the data [24] on liposome uptake by monocytes through recognition of opsonins on the surface of the lipid bilayer.

Analysis of the average fluorescence intensity of consumed liposomes for positive populations of monocytes and neutrophils (*Fig. 4*) allows us to draw the following conclusions. The presence of CMG (regardless of the presence of the prodrug) promoted internalization by monocytes. Neutrophils, in general, tended to more actively phagocytize liposomes with the prodrug, regardless of the presence of an amphiphile in the bilayer.

MTX liposomes with the GM1 ganglioside exhibited unexpected properties. Despite the fact that after 30 min a greater number of monocytes accumulated MTX liposomes without amphiphiles than MTX liposomes with GM1 (*Fig. 3*), the average fluorescence intensity of monocytes with L-MTXDG-GM1 liposomes was higher than that of monocytes with the L-MTXDG liposomes without GM1 after 30 and 60 min of incubation (*Fig. 4*). The very presence of GM1 did not lead to more intense phagocytosis of liposomes by monocytes. However, monocytes consumed more liposomes with a combination of the prodrug and the ganglioside in a bilayer than any others. Presumably, in this case, monocyte receptors recognize the GM_1_ ganglioside molecules or the plasma proteins associated with these liposomes.

**Fig. 5:**
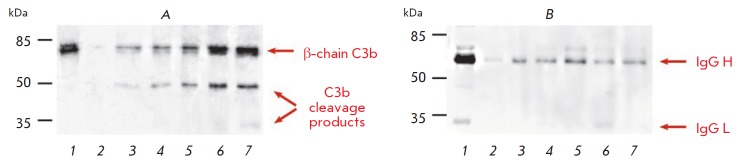
Identification of the proteins associated with liposomes using immunoblotting with antibodies to the component of the complement system C3 (*A*) and immunoglobulin G (*B*): *1 *– positive control, plasma diluted 1/500; *2 *– negative control, plasma after incubation with PBS, gel filtration, and delipidization; *3*–*7 *– liposome samples L (*3*), L-MTXDG (*4*), L-MTXDG-PI (*5*), L-MTXDG-GM1 (*6*), and L-MTXDG-CMG (*7*) after 15 min of incubation with human blood plasma, isolation of liposome–protein complexes, and delipidization

We decided to compare the binding of C3 and its plasma cleavage products to MTX liposomes containing various screening molecules. For this purpose, liposome preparations were incubated for 15 min in 80% blood plasma (as in the study of hemocompatibility, when the effect of MTX liposomes on C has been demonstrated [25]). Then, using liposome gel chromatography, liposome– protein complexes were isolated and the total amount of protein therein was determined. Plasma incubated with PBS was used to control the efficiency of separation of liposome–protein complexes from the bulk of unbound plasma proteins. As demonstrated by immunoblotting with antibodies to the C3 protein (*Fig. 5A*), MTX liposomes with GM1 and CMGPE cause significant fragmentation of the C component, with the formation of C3b cleavage products. In the case of L-MTXDG-PI and L-MTXDG samples, the amount of bound C3 and its cleavage products is noticeably lower. According to the cytometry data for 30 min (*Fig. 4A*), it is the L-MTXDG-GM1 and L-(MTXDG)-CMG liposomes that are accumulated the most by monocytes. Moreover, there was no increase in phagocytosis of CMG liposomes by monocytes due to the presence of MTXDG in the membrane, in contrast to GM1 liposomes (*Fig. 4A*). Apparently, it is the CMG residue that determines the interaction with proteins (and the subsequent interaction with monocytes), since its structure is exposed on the surface of liposomes to a greater extent than the MTX residue.

Immunoblotting with antibodies to IgG (*Fig. 5B*) did not reveal differences between liposome variants and the level of immunoglobulin binding was significantly lower than that in the case of the C3 factor.

Our results show that the presence of the methotrexate prodrug in the liposome membrane, in general, enhances their phagocytosis by both monocytes and neutrophils (the latter absorb liposomes significantly less actively). Introduction of PI into MTX liposomes practically does not affect their consumption by cells, and the CMG–lipid conjugate rather contributes to the accumulation of liposomes by monocytes. The GM1 ganglioside has a dual effect. Presumably, it shields MTX liposomes from phagocytosis by one population of monocytes (hence a smaller proportion of positive monocytes after 30 min of incubation), but it increases the efficiency of uptake by another monocyte population, probably with increased expression of C3b-binding receptors, which is reflected in the increase in the average fluorescence intensity of monocytes. The pattern of consumption of various liposomes by monocytes and neutrophils is summarized in the *Table*.

**Table:** Difference in the uptake of liposomes of various compositions by subpopulations of leukocytes upon 60 min incubation of the liposomes with whole blood^*^

Liposomes	Monocytes				Neutrophils		
	positive cells, %		X-mean		positive cells, %	X-mean	
	16–20	55–65	3	4–5	≥ 90	1–2	2–3
L	+		+		+	+	
L-PI	+		+		+	+	
L-GM_1_	+		+		+	+	
L-CMG	+			+	+	+	
L-MTXDG		+		+	+		+
L-MTXDG-PI		+		+	+		+
L-MTXDG-GM_1_		+		+^**^	+		+
L-CMG		+		+	+		+

^*^Data of Figs. 3 and 4 are summarized.

^**^X-mean average value is 7.

**Fig. 6:**
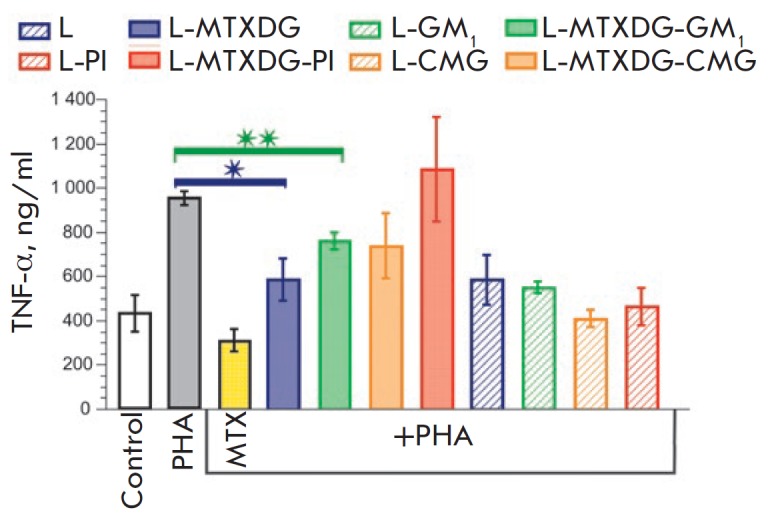
The effect of methotrexate and liposomes on the production of TNF-α by activated donor blood leukocytes. Mean values ± SD of two independent experiments are presented, each in two repeats; * *p* < 0.005, ** *p* < 0.0005

Lymphocytes are the main cells of the immune system that provide humoral and cellular immunity. According to our data, lymphocytes accounted for approximately 40% of the entire population of blood leukocytes (*Fig. 2*). We did not observe any uptake of liposomes by lymphocytes during 1 h of incubation with blood. Moreover, according to [39, 40], after 3–5 h of incubation, the lymphocytes also did not accumulate liposomes (of a different composition), notwithstanding the fact that in the cited papers not only internalized liposomes, but also those adsorbed/bound on the cell surface were taken into account. We found it interesting to determine how our liposomes affected lymphocyte functions. Given the fact that MTX liposomes are intended for the treatment of diseases accompanied by inflammatory processes, including oncological ones, we chose a model of activated leukocytes. The effect of liposomes on lymphocytes was evaluated by the change in the level of production of tumor necrosis factor alpha (TNF-α). Blood leukocytes were activated by a mitogen phytohemagglutinin (PHA), which preferably induces the production of TNF-α in T cells (for example, [45]). To activate leukocytes, diluted blood of healthy donors was incubated with PHA for 4 h then methotrexate or MTX liposome samples were added at equimolar concentrations (close to those in cytometry experiments) and incubated for another 4 h. The level of TNF-α in the culture fluid was determined by ELISA. The results are presented in *Fig. 6*.

The effect of MTX, as expected (for example, [46]), led to a pronounced suppression of cytokine production by the activated leukocytes (*Fig. 6*). Incorporation of MTXDG into the liposomes of ePC (L-MTXDG) reduced the effect of methotrexate. Obviously, MTX liposomes need time to be internalized by activated lymphocytes and undergo processing to release MTX. Indeed, in the culture of proliferating T lymphoblastoid cells, the cytotoxicity of intact MTX was an order of magnitude higher than the cytotoxicity of L-MTXDG-PI liposomes after 48 h of incubation [11]. Introduction of various protective molecules in MTX liposomes resulted in even further decrease in TNF-α production under the effect of the liposomes, to a varying extent (*Fig. 6*). MTX liposomes with PI practically did not inhibit the production of TNF-α, while L-MTXDG-GM1 liposomes suppressed cytokine production, yet to a lesser extent than MTX liposomes without the ganglioside (*p* < 0.05, or about 30% versus 60% compared to the level of production of TNF-α by intact cells). The average TNF-α level under the effect of L-MTXDG-CMG liposomes was the same as that in the case of L-MTXDG-GM1, although it did not differ significantly from the production of cytokine by the PHA-activated control cells (*Fig. 6*). The results can be explained in combination with the immunoblotting data (*Fig. 5A*): MTX liposomes with GM1 or CMGPE carry more protein ligands on their surface capable of binding to receptors on lymphocytes than liposomes with PI; therefore, they are internalized and inhibit cytokine production more actively. In addition, the inhibitory effect of L-MTXDG-GM1 liposomes may be due to specific interactions of ganglioside GM1. It can be assumed that GM1 is presented on the surface of MTX liposomes in such a way that it is able to bind, for example, galectins, extracellular matrix glycoproteins secreted by activated immunocompetent cells (e. g., galectin-1 is the main GM1 ganglioside receptor [47]).

Interestingly, all prodrug-free liposome samples also suppressed the production of TNF-α, but not as much as methotrexate (*Fig. 6*). Liposomes as such, without a cytostatic agent, apparently bind to receptor complexes on the cell surface, which can lead to inhibition of some signaling pathways of cytokine production in the case of activated lymphocytes or vice versa induce cytokine production by intact cells through activation of other signaling pathways. For example, it has been shown that phosphatidylcholine (along with α-galactosylceramide) is able to bind intracellularly the CD1d glycoprotein present on the cell surface and activate the so-called phospholipid-reactive T cells, which is an important regulatory mechanism for maintaining immune homeostasis between different pools of lipidreactive T cells [48]. Indeed, we observed an activation of TNF-α production by inactive leukocytes under the effect of simple liposomes of egg phosphatidylcholine (data not shown). Obviously, the effect of MTX liposomes of various compositions on activated leukocytes is mediated by a complex of factors: the number of liposome-associated proteins, the surface charge of liposomes (zeta potential of L-MTXDG-PI liposomes is –53 mV [25]; the values for MTX liposomes with GM1 or CMG the negative value should be even greater), the effect of phospholipids as such, and other molecular mechanisms.

## CONCLUSION

In accordance with existing views, the results of this work show that blood monocytes are the main phagocytes of nanosized liposomes of various compositions. Resting lymphocytes do not accumulate liposomes. Introduction of a methotrexate prodrug into the liposome membrane accelerates their phagocytosis and increases their uptake level by monocytes, regardless of the presence of protective amphiphilic molecules – phosphatidylinositol, ganglioside GM1, or CMGPE conjugate – in the membrane. All MTX liposome variants cause fragmentation of the central component of the complement system C3 and carry C3b cleavage products on the surface, which contributes to their capture by monocytes. Activation of the complement system can be caused by distortions induced by voluminous MTX moieties into the liposome surface structure, as well as by exocyclic aromatic amino functions and free α-COOH groups (amino (and hydroxy) groups of the moieties arranged in a certain way on the liposome surface can cause C activation by nucleophilic attack of the internal thioether bond in the C3b fragment [49]). It was unexpected that molecules carrying bulky negatively charged residues of a pentasaccharide or carboxymethylated oligoglycine did not have a screening effect, and that the corresponding MTX liposomes exhibited the highest level of C3 binding and fragmentation. A possible consequence of this was an increase in their effect on the function of activated lymphocytes compared with MTX liposomes containing phosphatidylinositol, although in general the results of these experiments are difficult to interpret unambiguously. In conclusion, it should be stated that the behavior of MTX liposomes in relation to blood leukocytes is determined to a greater extent by the methotrexate residue itself, and not by other components of the liposome bilayer. Some modulation of the effect of MTX liposomes on activated leukocytes can be achieved by introducing various screening molecules into the bilayer.

## Abbreviations

MTX: methotrexate
MTXDG: 1,2-rac-dioleoylglycerol ester conjugate of MTX
C: complement
ePC: egg yolk phosphatidylcholine
PI: phosphatidylinositol
CMGPE: N-carboxymethylated oligoglycine conjugate with phosphatidylethanolamine
TMB-PC: 1,3,5,7-tetramethyl-BODIPY-labeled phosphatidylcholine
PHA: phytohemagglutinin
L: ePC liposomes
L-MTXDG: ePC–MTXDG, 9 : 1, liposomes
L-MTXDG-PI: ePC–MYXDG–PI, 8 : 1 : 1, liposomes
L-MTXDG-CMG: ePC–MTXDG–CMG, 8 : 1 : 1, liposomes
L-MTXDG-GM1: ePC–MTXDG–GM1, 8 : 1 : 1, liposomes

## References

[1] (2019). World Health Organization. 20th WHO Model List of Essential Medicines (March 2017, amended August 2017). Accessed July 09, 2019.

[2] Weinblatt M.E. (2013). Trans. Am. Clin. Climatol. Assoc..

[3] McGuire J. (2003). Curr. Pharm. Des..

[4] Pui C.H. (1995). N. Engl. J. Med..

[5] Abolmaali S.S., Tamaddon A.M., Dinarvand R. (2013). Cancer. Chemother. Pharmacol..

[6] Rajitha P., Biswas R., Sabitha M., Jayakumar R. (2017). Curr. Pharm. Des..

[7] Liu L., Hu F., Wang H., Wu X., Eltahan A.S., Stanford S., Bottini N., Xiao H., Bottini M., Guo W. (2019). ACS Nano..

[8] Ding Q., Si X., Liu D., Peng J., Tang H., Sun W., Rui M., Chen Q., Wu L., Xu Y. (2015). J. Control. Release..

[9] Zucker D., Marcus D., Barenholz Y., Goldblum A. (2009). J. Control. Release..

[10] Vodovozova E.L., Kuznetsova N.R., Kadykov V.A., Khutsyan S.S., Gaenko G.P., Molotkovsky Y.G. (2008). Nanotechnol. Russia. Nanotechnol. Russia..

[11] Kuznetsova N., Kandyba A., Vostrov I., Kadykov V., Gaenko G., Molotkovsky J., Vodovozova E. (2009). J. Drug. Deliv. Sci. Technol..

[12] Alekseeva A.S., Moiseeva E.V., Onishchenko N.R., Boldyrev I.A., Singin A.S., Budko A.P., Shprakh Z.S., Molotkovsky J.G., Vodovozova E.L. (2017). Int. J. Nanomedicine..

[13] Barenholz Y. (2012). J. Control. Release..

[14] Allen T.M., Cullis P.R. (2013). Adv. Drug Deliv. Rev..

[15] Bulbake U., Doppalapudi S., Kommineni N., Khan W. (2017). Pharmaceutics..

[16] Gustafson H.H., Holt-Casper D., Grainger D.W., Ghandehari H. (2015). Nano Today..

[17] Betker J.L., Jones D., Childs C.R., Helm K.M., Terrell K., Nagel M.A., Anchordoquy T.J. (2018). J. Control. Release..

[18] Lasic D.D., Papahadjopoulos D. (1995). Science..

[19] Ilinskaya A.N., Dobrovolskaia M.A. (2016). Toxicol. Appl. Pharmacol..

[20] Palchetti S., Colapicchioni V., Digiacomo L., Caracciolo G., Pozzi D., Capriotti A.L., La Barbera G., Laganà A. (2016). Biochim. Biophys. Acta – Biomembr..

[21] Szebeni J., Muggia F., Gabizon A., Barenholz Y. (2011). Adv. Drug Deliv. Rev..

[22] Yang Q., Lai S.K. (2015). Wiley Interdiscip. Rev. Nanomed. Nanobiotechnol..

[23] Tenzer S., Docter D., Kuharev J., Musyanovych A., Fetz V., Hecht R., Schlenk F., Fischer D., Kiouptsi K., Reinhardt C. (2013). Nat. Nanotechn..

[24] Bros M., Nuhn L., Simon J., Moll L., Mailänder V., Landfester K., Grabbe S. (2018). Front. Immunol..

[25] Kuznetsova N.R., Sevrin C., Lespineux D., Bovin N.V., Vodovozova E.L., Mészáros T., Szebeni J., Grandfils C. (2012). J. Control. Release..

[26] Tretiakova D.S., Onishchenko N.R., Vostrova A.G., Vodovozova E.L. (2017). Russ. J. Bioorg. Chem..

[27] Kuznetsova N.R., Vodovozova E.L. (2014). Biochemistry (Mosc.)..

[28] Vodovozova E.L., Gaenko G.P., Bobrikova E.S., Pazynina G.V., Molotkovskii Y.G. (2007). Pharm. Chem. J..

[29] Boldyrev I.A., Zhai X., Momsen M.M., Brockman H.L., Brown R.E., Molotkovsky J.G. (2007). J. Lipid Res..

[30] Jaye D.L., Geigerman C.M., Fuller R.E., Akyildiz A., Parkos C.A. (2004). J. Immunol. Methods..

[31] Markwell M., Haas S., Bieber L. (1978). Anal. Biochem..

[32] Dos Santos N., Allen C., Doppen A.M., Anantha M., Cox K., Gallagher R.C., Karlsson G., Edwards K., Kenner G., Samuels L. (2007). Biochim. Biophys. Acta..

[33] Laemmli U.K. (1970). Nature.

[34] Shevchenko A., Wilm M., Vorm O., Mann M. (1996). Anal. Chem..

[35] Gabizon A., Papahadjopoulos D. (1988). Proc. Natl. Acad. Sci. USA..

[36] Muller M., Zschornig O., Ohki S., Arnold K. (2003). J. Membrane Biol..

[37] Allen T.M., Hansen C., Rutledge J. (1989). Biochim. Biophys. Acta..

[38] Tretiakova D., Onishchenko N., Boldyrev I., Mikhalyov I., Tuzikov A., Bovin N., Evtushenko E., Vodovozova E. (2018). Colloids Surf. B Biointerfaces..

[39] Karathanasis E., Geigerman C.M., Parkos C.A., Chan L., Bellamkonda R.V., Jaye D.L. (2009). Ann. Biomed. Eng..

[40] Münter R., Kristensen K., Pedersbæk D., Larsen J.B., Simonsen J.B., Andresen T.L. (2018). Nanoscale..

[41] Francian A., Mann K., Kullberg M. (2017). Int. J. Nanomedicine..

[42] Bisso P.W., Gaglione S., Guimarães P.P.G., Mitchell M.J., Langer R. (2018). ACS Biomater. Sci. Eng..

[43] Hed J. (1977). FEMS Lett..

[44] Roitt I., Brostoff J., Male D. (1998). Immunology, 5th ed. London– Philadelphia–St. Louis–Sydney–Tokyo: Mosby, 1998. 423 p..

[45] Ferrante A., Staugas R.E., Rowan-Kelly B., Bresatz S., Kumaratilake L.M., Rzepczyk C.M., Adolf G.R. (1990). Infect. Immun..

[46] Hildner K., Finotto S., Becker C., Schlaak J., Schirmacher P., Galle P.R., E. Marker-Hermann E., Neurath M.F. (1999). Clin. Exp. Immunol..

[47] Kopitz J., von Reitzenstein C., Burchert M., Cantz M., Gabius H.J. (1998). J. Biol. Chem..

[48] Halder R.C., Tran C., Prasad P., Wang J., Nallapothula D., Ishikawa T., Wang M., Zajonc D.M., Singh R.R. (2019). Eur. J. Immunol..

[49] Janssen B.J.C., Christodoulidou A., McCarthy A., Lambris J.D., Gros P. (2006). Nature.

